# Vitamin D Deficiency: Effects on Oxidative Stress, Epigenetics, Gene Regulation, and Aging

**DOI:** 10.3390/biology8020030

**Published:** 2019-05-11

**Authors:** Sunil J. Wimalawansa

**Affiliations:** Professor of Medicine, Endocrinology & Nutrition, Cardio Metabolic and Endocrine Institute, NJ 08873, USA; suniljw@hotmail.com

**Keywords:** 25(OH)D, 1,25(OH)_2_D, aging, cytokines, inflammation, morbidity and mortality, prevention, reactive oxygen species, ultraviolet

## Abstract

Recent advances in vitamin D research indicate that this vitamin, a secosteroid hormone, has beneficial effects on several body systems other than the musculoskeletal system. Both 25 dihydroxy vitamin D [25(OH)_2_D] and its active hormonal form, 1,25-dihydroxyvitamin D [1,25(OH)_2_D] are essential for human physiological functions, including damping down inflammation and the excessive intracellular oxidative stresses. Vitamin D is one of the key controllers of systemic inflammation, oxidative stress and mitochondrial respiratory function, and thus, the aging process in humans. In turn, molecular and cellular actions form 1,25(OH)_2_D slow down oxidative stress, cell and tissue damage, and the aging process. On the other hand, hypovitaminosis D impairs mitochondrial functions, and enhances oxidative stress and systemic inflammation. The interaction of 1,25(OH)_2_D with its intracellular receptors modulates vitamin D–dependent gene transcription and activation of vitamin D-responsive elements, which triggers multiple second messenger systems. Thus, it is not surprising that hypovitaminosis D increases the incidence and severity of several age-related common diseases, such as metabolic disorders that are linked to oxidative stress. These include obesity, insulin resistance, type 2 diabetes, hypertension, pregnancy complications, memory disorders, osteoporosis, autoimmune diseases, certain cancers, and systemic inflammatory diseases. Vitamin D adequacy leads to less oxidative stress and improves mitochondrial and endocrine functions, reducing the risks of disorders, such as autoimmunity, infections, metabolic derangements, and impairment of DNA repair; all of this aids a healthy, graceful aging process. Vitamin D is also a potent anti-oxidant that facilitates balanced mitochondrial activities, preventing oxidative stress-related protein oxidation, lipid peroxidation, and DNA damage. New understandings of vitamin D-related advances in metabolomics, transcriptomics, epigenetics, in relation to its ability to control oxidative stress in conjunction with micronutrients, vitamins, and antioxidants, following normalization of serum 25(OH)D and tissue 1,25(OH)_2_D concentrations, likely to promise cost-effective better clinical outcomes in humans.

## 1. Introduction

Vitamin D is a micronutrient that is metabolized into a multifunctional secosteroid hormone that is essential for human health. Globally, its deficiency is a major public health problem affecting all ages and ethnic groups; it has surpassed iron deficiency as the most common nutritional deficiency in the world. The increasing prevalence of vitamin D deficiency and its associated complications are prominent in countries furthest from the equator. However, incidence is also high among those who live within 1,000 km of the equator (e.g., in Sri Lanka, India, and Far Eastern, Middle Eastern, Central American, and Persian Gulf countries) because of a combination of climatic conditions, ethnic and cultural habits, and having darker skin color [1,2,3,4].

Most of the vitamin D the human body requires can be generated by an individual’s exposure to summer-like sunlight, with dietary sources playing a supporting role when sunlight exposure is limited or ineffective for vitamin D production. Despite the above, more than 50% of the population in the group of countries mentioned has vitamin D deficiency [5,6]. If effective public health guidelines are implemented, vitamin D deficiency can be easily and cost-effectively treated and prevented, saving millions of dollars and lives. Although excessive sun exposure does not cause hypervitaminosis D, it can cause other harm because of dermal cell DNA damage [7,8,9]. Thus, guidelines for safe sun exposure are needed for each country.

During the past decade, many advances in the understanding of the physiology and biology of vitamin D and its receptor ecology have emerged [10]. Vitamin D metabolism and functions are modulated by many factors. Accumulating evidence supports biological associations of vitamin D with disease risk reduction and improved physical and mental functions. The field is rapidly advancing, including the knowledge of the physiology of vitamin D-vitamin D receptor (VDR) interactions and the biology and metabolism of vitamin D and their effects on vitamin D axis and gene polymorphisms [11]. Together these data have facilitated our understanding of new pathways to intervene to prevent and treat human diseases.

However, what is lacking is the adequately powered, conducted for sufficient duration, well-designed randomized controlled clinical studies (RCTs) conducted in an unbiased manner with the nutrient vitamin D as the key intervention and having predefined hard endpoints/primary outcomes [12]. Moreover, such studies must recruit persons with vitamin D deficiency [i.e., serum 25(OH)D concentrations less than 20 ng/mL (50 nmol/L)] and achieve a pre-determined target serum 25(OH)D concentration through daily oral administration and/or safe exposure to ultraviolet rays; not merely by relying on oral administered doses. The goal of this review is to explore the effects from vitamin D-modulated gene interactions and the effects of hypovitaminosis-induced, mitochondria-based oxidative stress on aging.

### 1.1. Extrarenal Generation of 1,25(OH)_2_D

The active form of vitamin D, 1,25-dihydroxyvitamin D [1,25(OH)_2_D], is generated not only in renal tubular cells (endocrine functions as a hormone) but also in extrarenal target tissue cells, providing autocrine and paracrine functions. However, because it remains within the target tissue cells, the intra-cellular concentrations achieved are unclear. In addition, the catabolic activity of 24-hydroxylase in target tissues plays an important part in regulating both 25-hydroxy vitamin D [25(OH)D; calcidiol], and 1,25(OH)_2_D (calcitriol) concentrations and their availability.

The amounts of 1,25(OH)_2_D generated in renal tubules and target cells can vary from person to person and day to day and are hard to quantify. Although the calcitriol in the circulation is modulated by parathyroid hormone (PTH) and serum ionized calcium concentrations [13], the intracellular content is regulated largely through serum 25(OH)D availability and calcidiol and calcitriol catabolism through hydroxylation at the molecular positions C-24 and C-23 by a specific 24-hydroxylase (CYP24A1) [14].

### 1.2. Excess Sun Exposure Does Not Cause Hypervitaminosis D

After exposure to ultraviolet B (UVB) rays, dermal cells actively synthesize vitamin D. As a feedback mechanism, excess precursors produced are catabolized within the dermal cells by the same UVB rays. In addition, skin also contains the inactivating enzyme 24-hydroxylase, which also prevents over-production of vitamin D by 24-hydroxylation of vitamin D [15]. This process of homeostasis is regulated by UVB, PTH, and serum ionized calcium concentrations [16]. When an individual is overexposed to ultraviolet rays, the mentioned built-in protective mechanism prevents excessive retention of vitamin D in the skin. Therefore, sun exposure does not raise serum 25(OH)D to pathological levels, cause hypervitaminosis D or its complications, such as hypercalcemia.

In addition, vitamin D synthesized in the skin from UVB exposure in excess of need is catabolized in part through 20-hydroxylation by the cholesterol side chain cleavage enzyme CYP11A1 [17]. Thus, there are multiple intrinsic mechanisms present to prevent excess vitamin D from reaching the circulation. The efficiency of vitamin D synthesis in the skin is affected by many factors, including the density of melanin pigment, condition of the skin and age, and the use of sunscreen and UV-blocking makeup, creams, and ointments and clothing. In addition, older age or scarred skin, as well as time of day or year and the duration of sun exposure affect vitamin D synthesis in the skin [18,19,20,21].

Although solar radiation is the source of vitamin D generation in the skin, excessive exposure, particularly in those who are genetically vulnerable, may cause skin cancers [22,23,24]. On the other hand, optimal vitamin D status protects against several types of internal cancers, melanoma, and several other diseases. Therefore, one needs to balance sun exposure in favor of benefits while avoiding potential harmful effects [25,26,27].

## 2. Vitamin D and Gene Regulation

1,25(OH)_2_D regulates many genes within the human genome [28]. Tissue vitamin D concentrations and its receptor gene polymorphisms, not only influence mechanism of actions and modulate second messenger systems, but also modulate the ability of the ligand-bound VDR to bind to vitamin D response elements (VDREs) on promoter regions in genes and initiate second messenger systems [29]. The National Human Genome Research Institute (NHGRI) launched a public research consortium, ENCODE [Encyclopedia of DNA Elements; http://www.genome.gov/10005107] to answer pertinent questions [30].

ENCODE has demonstrated the genome-wide actions of 1,25(OH)_2_D_3_ on the formation rates of proteins, such as the insulator protein CTCF (transcriptional repressor CTCF; 11-zinc finger protein, CCCTC-binding factor, etc.) and the VDR [31,32]. These findings suggest the presence of numerous functional VDRE regions across the human genome [29,33]. In addition, it has been suggested that the expression of these genes could be used as biomarkers for different actions of vitamin D in varied tissues and cells and assessment of vulnerabilities [34].

Hormone, 1,25(OH)_2_D modulates cell proliferation through direct and indirect pathways. For example, vitamin D inhibits the pathways related to transcription factor NF-κB [35]. People with chronic non-communicable diseases, such as cardiovascular disease, type 2 diabetes, autoimmune diseases, arthritis, and osteoporosis are reported to have chronically elevated NF-κB [36]. NF-κB enhances the oxidative stress and cellular responses to inflammation and injury; including following head injury [37]. Whereas, 1,25(OH)_2_D (calcitriol) suppresses NF-κB and thereby reduces chronic diffuse somatic inflammation [38,39]. In addition, calcitriol also reduce cell proliferation and enhance cell differentiation—key anti-cancer effects of vitamin D [40]. 

### 2.1. Epigenetic Mechanisms Influence Cancer Genesis

Epigenetic mechanisms influence cancer genesis, growth, dissemination, and aging phenomena [41,42,43,44]. For example, the epigenetic modifications of VDR–1,25(OH)_2_D effects can be mediated through complex processes involving CYP27A1 and CYP27B1 and via the vitamin D-catabolizing enzyme CYP24 [14,44]. These actions can be favorably influenced by modifications of VDREs across the genome modulated by both histone acetylases and deacetylases [28,29,45].

Epigenetic regulation of vitamin D metabolism influences several physiological mechanisms and modulate outcomes of some human diseases. Example of diseases include adenocarcinoma of the lung [44], specific gene mutations in Asians with advanced non-small cell lung cancer [41], and genetic alterations in the effectiveness of systemic therapy for lung cancer induced by cigarette smoking [42]. In severely obese children, low 25(OH)D concentrations are associated with increased markers of oxidative and nitrosative stress, inflammation, and endothelial over-activation [46].

CYP27B1-mediated target tissue production of 1,25(OH)D is critically important for the paracrine and autocrine functions of calcitriol to obtain the full biological potential of vitamin D. Taken together, the benefits of having adequate serum 25(OH)D concentrations and maintaining vitamin D repletion in the long run and considering the overall health benefits of vitamin D, there is an urgent need to create national policies to combat hypovitaminosis D. The savings derived from reducing the risks and severity of infectious and parasitic diseases alone would pay-off the cost of this public health approach. This can be achieved through targeting to raise the population serum 25(OH)D concentration, leading to a tangible positive impact on humans and on the economy.

### 2.2. Epigenetics and Molecular Genetics of Vitamin D

Molecular and genetic studies confirm that vitamin D also modulates risks of several other human diseases, including autoimmune disorders such as multiple sclerosis [47]. Although the predominant cause of cancer is modulation of the underlying metabolic abnormalities through genes, such as p53 and c-myc modifying the metastatic risks, the responsiveness to therapy is in part determined by epigenetic modifications of genes.

Tumor-related key metabolic abnormalities include imbalance between glucose fermentation and oxidative phosphorylation (under aerobic and anaerobic conditions—the Warburg effect); dysregulation of metabolic enzymes, such as pyruvate kinase, fumarate hydratase, and succinate dehydrogenase; isocitrate dehydrogenase mutations; and alterations of gene expression levels linked to tumorigenesis that are influenced by the vitamin D status [48].

Examples related to activity of the vitamin D axis include epigenetic changes that affect the expression of the CYP24A1 gene and VDR polymorphisms. Although epigenetic enhancement can occur through methylation and repression by histone-modifications of DNA, vitamin D markedly influences the regulation of cell replication [42,44]. This substantiates targeting of CYP24A1 to optimize the antiproliferative effects of 1,25(OH)_2_D in a target-specific manner [49].

In addition, gene activation following the interaction of 1,25(OH)_2_D with VDR is important for mitochondrial integrity and respiration, and many other physiological activities. Moreover, the vitamin D signaling pathway plays a central role in protecting cells from elevated mitochondrial respiration and associated damage and overproduction of reactive oxygen species (ROS), which can lead to cellular and DNA damage [50].

## 3. Vitamin D–Oxidative Stress

1,25(OH)_2_D is involved in many intracellular genomic activities and biochemical and enzymatic reactions, whereas 25(OH)D concentrations are important in overcoming inflammation, the destruction of invading microbes and parasites, the minimization of oxidative stress following the day-to-day exposure to toxic agents, and controlling the aging process [51,52].

**Figure 1:**
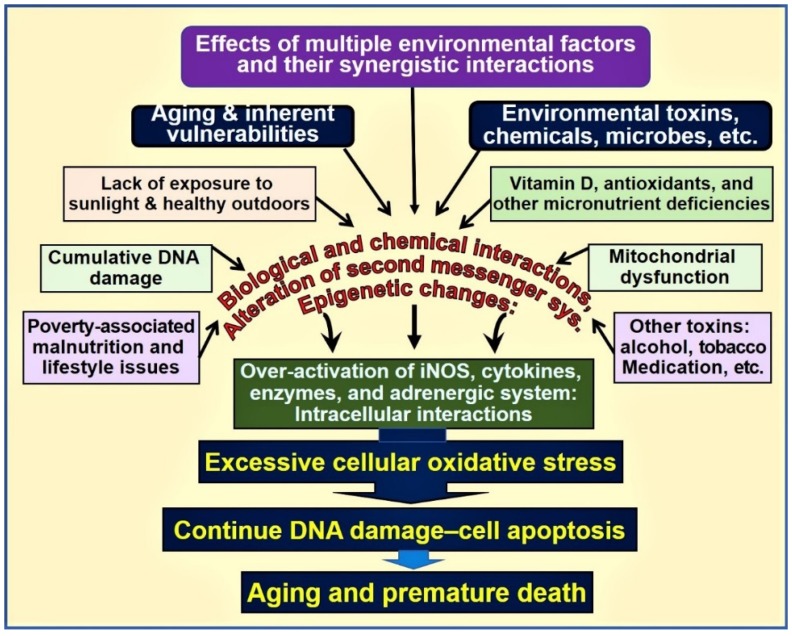
Environmental, microbial, biological and chemical interactions that modify the DNA and mitochondrial functions and epigenetics, which modifies the aging process. Vitamin D deficiency is one of the factors that enhances this oxidative-stress cycle and accelerating premature cell death [abbreviations used: DNA = deoxyribonucleic acid; iNOS = inducible nitric oxide enzyme].

For example, the presence of a physiologic 25(OH)D concentration enhances the expression of the nuclear factor, erythroid-2(Nf-E2)-related factor 2(Nrf2) [53,54,55] and enhances Klotho, a phosphate regulating hormone and also an antiaging protein [56,57]. It also facilitates protein stabilization [11]. Klotho also regulates cellular signaling systems, including the formation of antioxidants [58]. Consequently, in mice, functional abnormalities of the Klotho gene or removal of it through gene knock-out procedures induce premature aging syndrome [59]. In animal studies, inefficient FGF23 and/or Klotho expression have shown to cause premature aging. Figure 1 is a schematic representation of various key factors and their interactions that influence aging and death. 

### Influences of Vitamin D on Oxidative Stress

When vitamin D status is adequate, many of the intracellular oxidative stress-related activities are downregulated. Having suboptimal concentrations of serum 25(OH)D fails to subdue oxidative stress conditions, augment intracellular oxidative damage and the rate of apoptosis. The intracellular Nrf2 level is inversely correlated with the accumulation of mitochondrial ROS [51,60] and the consequent escalation of oxidative stress. Thus, Nrf2 plays a key role in protecting cells against oxidative stress; this is modulated by vitamin D [61,62].

In addition, vitamin D supports cellular oxidation and reduction (redox) control by maintaining normal mitochondrial functions [63,64,65]. Loss in the redox control of the cell cycle may lead to aberrant cell proliferation, cell death, the development of neurodegenerative diseases, and accelerated aging [65,66,67,68]. Peroxisome proliferator-activated receptor-coactivator 1α (PGC-1α) is bound to mitochondrial deacetylase (SIRT3). PGC-1α directly couples to the oxidative stress cycle [69] and interacts with Nrf2. This complex regulates the expression of SIRT3; this process is influenced by vitamin D metabolites [70]. In addition, the activation of the mitochondrial Nrf2/PGC-1α-SIRT3 path is dependent on intracellular calcitriol concentrations. 

Calcitriol has overarching beneficial effects in upregulating the expression of certain antioxidants and anti-inflammatory cytokines [71], thereby protecting the tissues from toxins, micronutrient deficiency-related abnormalities, and parasitic and intracellular microbe-induced harm [72]. It regulates ROS levels through its anti-inflammatory effects and mitochondrial-based expression of antioxidants through cell-signaling pathways [67,73].

## 4. Role of Vitamin D in Neutralization of Toxins and Aging-Related Compounds

Following 1,25(OH)_2_D—VDR interaction, the transcription factor Nrf2 translocates from the cytoplasm to the nucleus. Nrf2 activates the expression of several genes that have antioxidant activity [52,54,67]. When Nrf2 activity is insufficient, risks from oxidative stress-related tissue damage increases [61,74]. The resultant excessive ROS formation by dysregulated mitochondria leads to a pathologic oxidative stress cycle, a key cause of toxin-induced and age-related cell death [68,75,76].

Meanwhile, the Nrf2 activity in part is controlled by the cytosolic protein Keap1 [77], another transcription factor and a negative regulator of Nrf2 [55,61]. Keap1 also controls the subcellular distribution of Nrf2 that correlates with its antioxidant activity [53,54]. When confronted with intracellular oxidative and/or electrophilic stresses, as a protective mechanism, the Nrf2-antioxidant response path is activated. This response enhances gene transcription and translation of protein products that are necessary to eliminate and/or neutralize toxins, ROS, and cumulating aging-related products through conjugation [78,79].

### 4.1. The Concept and the Process of Aging

Aging generally refers to the biological process of growing older, also known as cellular senescence, and is a complex process. Advancing age is, especially after adulthood, is associated with a gradual decline of physiological functions and capacities [80]. Aging has also been quantified from mortality curves using mathematical modeling; for example, by using Gompertz equation m(t) = Ae^Gt^, for which, m(t) = the mortality rate as a function of time or age (*t*); A = extrapolated constant to birth or maturity; G = the exponential (Gompertz) mortality rate coefficient] [79]. 

Moreover, efficiency and the functions of the body decline after sexual maturity, suggesting a connection between the aging process after fulfilling the procreation needs. Most age-related functions are irreversible, in part due to accumulation of oxidative stress-related toxic products, methylation of DNA, and mitochondrial damage, leading to reduced viability of cells and consequent accelerated cell death [81]. There is also a parallel decline in the immune system functions (i.e., immune-senescence) and an increase in inflammation, demonstrable with increased circulating pro-inflammatory cytokines [82,83]. These are likely to contribute to several age-related disorders, such as Alzheimer’s disease, cardiovascular and pulmonary diseases, and susceptibility to autoimmunity and infections [82,83].

Many bodily functions slow with aging, including response and reaction time; access to and the capacity of memory; pulmonary, gastrointestinal, and cardiovascular capacities; and even the ability to generate vitamin D in the skin. While age is perhaps the strongest risk factors for death, age-related disorders are the number one cause of death among the adults. This scenario is aggravated in the presence of vitamin D deficiency.

Chronic hypovitaminosis D is associated with cardiovascular and metabolic dysfunctions and premature deaths [84], even among children [85]. Overall data suggest that vitamin D deficiency could be considered an important comorbidity or a risk factor for premature death [84,85,86,87]. In fact, inverse relationships have been reported with vitamin D adequacy, with reduced all-cause mortality [88,89,90], and cancer [90,91,92,93].

### 4.2. Effects of Vitamin D on Apoptosis and Aging

The generalized inflammatory process is known to cause cellular damage and increase apoptosis [94], as in the case of interstitial tubular cell damage in chronic kidney disease, and thus is a part of the aging process [52,55,95]. In addition, hypovitaminosis D and dysfunctional mitochondrial activity increase inflammation [73,96,97]. Thus, the anti-inflammatory effects from having adequate, physiological vitamin D concentrations are important [95,98]. Hypovitaminosis D increases the expression of inflammatory cytokines [71,99] such as tumor necrosis factor-*α* (TNF-*α*), increasing the expression of the InsP3Rs and resulting in increased intracellular Ca^2+^ and accelerating cellular damage, apoptosis, and aging [66,75].

Many of the genes in the Klotho–Nrf2 regulatory system have multiple functions that are regulated by calcitriol [57,62,65]. These include, increasing intracellular antioxidant concentration, maintaining the redox homeostasis and, normal intracellular-reduced environment by removing excess ROS, and thereby down-regulating the oxidative stress [100]. In addition, the vitamin D-dependent expression of *γ*-glutamyl transpeptidase, glutamate cysteine ligase, and glutathione reductase contribute to the synthesis of the key redox agent glutathione (an essential antioxidant of low–molecular-weight thiol) [99,101].

Vitamin D also upregulates the expression of glutathione peroxidase that converts the ROS molecule H_2_O_2_ to water [101]. Vitamin D also effect the formation of glutathione through activation of the enzyme glucose-6-phosphate dehydrogenase [101]—which downregulates nitrogen oxide (NOx), a potent precursor for generating ROS that converts O_2_^−^ to H_2_O_2_ and upregulating superoxide dismutase (SOD). These vitamin D-related actions collectively reduce the burden of intracellular ROS.

Telomeres are repetitive DNA sequences that caps end of linear chromosomes protecting DNA molecules [102]. Aging is associated with shortening of telomeres, including in stem cells. The amount of telomerase present is gradually become too short to maintain its protective effects on DNA during cell division, and thus cell apoptosis. While vitamin D deficiency increases inflammation and the intracellular oxidative stress, the latter enhances the rate of telomere shortening during cell proliferation, resulting in genomic instability [36]. 

### 4.3. Hypovitaminosis D Leads to Deranged Mitochondrial Respiration

Activated vitamin D is an essential component for maintaining physiological respiratory chain activity in mitochondria, facilitating the generation of energy [103,104]. In addition, 25(OH)D regulates the expression of the uncoupling protein that is attached to the inner membrane of mitochondria that regulates thermogenesis [105,106,107]. Chronic vitamin D deficiency reduces the capacity of mitochondrial respiration through modulating nuclear mRNA [108,109,110]. The latter also downregulates the expression of complex I of the electron transport chain and thus reduces the formation of adenosine triphosphate (ATP) [67,75], another mechanism that increases cancer risks. Consequently, a low level of electron transport chain increases the formation of ROS and oxidative stress, a common phenomenon following acute and chronic exposure to toxins and many chronic diseases and seen in aging [66,111,112].

The accumulation of intracellular toxins and/or age-related products disrupts signaling pathways, including the G protein–coupled systems, caspases, mitochondria, and the death receptor-linked mechanisms, triggering cell apoptosis and causing premature cell death [113,114]. The process is aggravated by stimulating G proteins, leading to activation of downstream pathways, including protein kinase A and C (PKA and PKC), phosphatidylinositol 3 kinase (PI3-kinase), Ca^2+^ and MAP kinase-dependent systems, tyrosine phosphorylation [75,114], and work additively, aiding cancer genesis [93] and accelerating the aging process.

### 4.4. Calcitriol Protects Mitochondrial Functions

Toxins, chronic metabolic abnormalities, and the aging process are known to cause mitochondrial dysfunction [66,106,107,108,115]. Abnormal mitochondria produce suboptimal amounts of ATP while generating excess ROS, creating a vicious cycle of enhanced and persisting the effects from excessive oxidative stress [106,107,116]. These events cause DNA damage (and impair DNA repair systems), premature cell death, and accelerated aging [62,66]. Data are accumulating that suggest that mitochondrial dysfunction is likely fueled by sustained intracellular inflammation, as in the case with vitamin D deficiency [79,95,97,117].

Based on animal studies, researchers have reported that mitochondrial decay, a part of the aging process, can be slowed by micronutrient supplementation (e.g., by lipoic acid, acetyl carnitine, vitamin K, and vitamin D) and by boosting coenzyme levels through high doses of vitamin B, such as pantothenic acid [118]. The NAD^+^-dependent protein deacetylases sirtuins function as antiaging proteins that also neutralize excess ROS [11]. For example, sirtuin 1 (SIRT) is essential to maintaining normal mitochondrial functions; meanwhile, calcitriol and SIRT1 work synergistically to regenerate mitochondria [119,120]. Their actions facilitate the removal and neutralization of toxins and thereby reduce the rate and the effects of aging [121,122].

**Figure 2:**
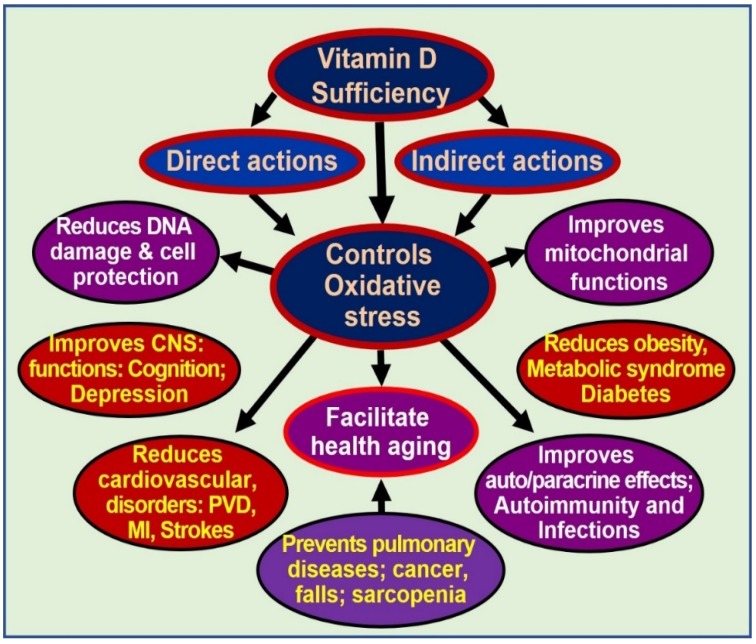
Oxidative stress is harmful to cells. Controlling oxidative stresses through vitamin D adequacy leads to cellular and organ protection and reduces the effects of aging [abbreviations used: CNS = central nervous system; DNA = deoxyribonucleic acid; MI = myocardial infarction; PVD = peripheral vascular diseases].

Dysfunctional mitochondria also have reduced intracellular Ca^2+^ buffering capacity, resulting in increased (and fluctuating) intracellular Ca^2+^ levels, which are cytotoxic and contribute to sustenance of several chronic diseases [108,115]. Sub physiological concentrations of calcitriol, at least in part, enhance and maintain oxidative stress, autophagy, inflammation, mitochondrial dysfunction, epigenetic changes, DNA damage, intracellular Ca^2+^, and generation and signaling of ROS. Therefore, sustained, adequate serum 25(OH)D concentrations should allow target tissues to keep many of these harmful processes under control [68,71,73]. Multiple benefits of controlling excessive oxidative stress are illustrated in Figure 2.

## 5. Discussion

The proper functioning of the vitamin D endocrine, paracrine, and autocrine systems is essential for many human physiological functions. Vitamin D deficiency, as determined by serum 25(OH)D concentrations of less than 30 ng/mL, is associated with increased risks of illnesses and disorders and increased all-cause mortality even among apparently healthy individuals, including those with normal serum 1,25(OH)_2_D. Some of the key functions of vitamin D include subduing oxidative stress and chronic inflammation and maintaining mitochondrial respiratory functions. Through its targeted mitochondrial activity and subduing of ROS through multiple mechanisms, vitamin D has key beneficial effects on controlling oxidative stress, inflammation, and energy metabolism.

Normal serum concentrations of both 25(OH)D and 1,25(OH)_2_D are essential for optimal cellular function and protect from the excessive oxidative stress-related DNA damage. However, increased risk for illnesses and reduced longevity can occur despite the presence of physiologic concentrations of calcitriol because this is not the only mechanism protecting cells from oxidative stress. Physiologic serum 25(OH)D and 1,25(OH)_2_D levels in target tissues allow exertion of the homeostatic modulatory effects on enzymatic reactions, mitochondrial activities, and functioning of optimal second messenger systems. These are essential parts of the actions of vitamin D mediated through the mentioned mechanisms.

Vitamin D metabolism and functions are modulated by many factors, including physical activities and lifestyles, certain medications, environmental pollutants, and epigenetics, all of which also modify the balance between energy intake and expenditure through mitochondrial metabolic control [123]. For reductions in the incidence of diseases, longer-term maintenance of a steady state of the serum 25(OH)D concentration is necessary [124]. The minimal level is considered to be 30 ng/mL (50 nmol/L). 

After correction of vitamin D deficiency through loading doses of oral vitamin D (or safe sun exposure), adequate maintenance doses of vitamin D_3_ are needed. This can be achieved in approximately 90% of the adult population with vitamin D supplementation between 1000 to 4000 IU/day, 10,000 IU twice a week, or 50,000 IU twice a month [10,125]. On a population basis, such doses would allow approximately 97% of people to maintain their serum 25(OH)D concentrations above 30 ng/mL [19,126]. Others, such as persons with obesity, those with gastrointestinal disorders, and during pregnancy and lactation, are likely to require doses of 6,000 IU/day [127,128]. 

## Abbreviations

1,25(OH)_2_D	1,25-dihydroxyvitamin D
25(OH)D	25-hydroxy vitamin D
Nrf2	Nuclear factor erythroid 2 (Nf-E2)-related factor 2
PTH	Parathyroid hormone
ROS	Reactive oxygen species
UVB	Ultraviolet B
VDR	Vitamin D receptor
VDRE	Vitamin D response element
